# Efficient *In Vitro* Generation of IL-22-Secreting ILC3 From CD34^+^ Hematopoietic Progenitors in a Human Mesenchymal Stem Cell Niche

**DOI:** 10.3389/fimmu.2021.797432

**Published:** 2021-12-24

**Authors:** Sabrina B. Bennstein, Sandra Weinhold, Özer Degistirici, Robert A. J. Oostendorp, Katharina Raba, Gesine Kögler, Roland Meisel, Lutz Walter, Markus Uhrberg

**Affiliations:** ^1^ Institute for Transplantation Diagnostics and Cell Therapeutics, Medical Faculty, Heinrich-Heine University Düsseldorf, Düsseldorf, Germany; ^2^ Division of Pediatric Stem Cell Therapy, Clinic for Pediatric Oncology, Hematology and Clinical Immunology, Center for Children and Adolescence Health, Heinrich-Heine University Düsseldorf, Medical Faculty, Düsseldorf, Germany; ^3^ Technical University of Munich, School of Medicine, Klinikum rechts der Isar, Department of Internal Medicine III – Hematology and Oncology, Laboratory of Stem Cell Physiology, Munich, Germany; ^4^ Primate Genetics Laboratory, German Primate Center, Leibniz Institute for Primate Research, Göttingen, Germany

**Keywords:** CD34 cells^+^, mesenchymal stem cells, umbilical cord stem cells (UCSC), innate lymphoid cells (ILCs), ILC3, hematopoietic stem and progenitor cells, GMP—Good Manufacturing Practice

## Abstract

Innate lymphoid cells (ILCs) and in particular ILC3s have been described to be vital for mucosal barrier functions and homeostasis within the gastrointestinal (GI) tract. Importantly, IL-22-secreting ILC3 have been implicated in the control of inflammatory bowel disease (IBD) and were shown to reduce the incidence of graft-versus-host disease (GvHD) as well as the risk of transplant rejection. Unfortunately, IL-22-secreting ILC3 are primarily located in mucosal tissues and are not found within the circulation, making access to them in humans challenging. On this account, there is a growing desire for clinically applicable protocols for *in vitro* generation of effector ILC3. Here, we present an approach for faithful generation of functionally competent human ILC3s from cord blood-derived CD34^+^ hematopoietic progenitors on layers of human mesenchymal stem cells (MSCs) generated in good manufacturing practice (GMP) quality. The *in vitro*-generated ILC3s phenotypically, functionally, and transcriptionally resemble *bona fide* tissue ILC3 with high expression of the transcription factors (TF) RorγT, AHR, and ID2, as well as the surface receptors CD117, CD56, and NKp44. Importantly, the majority of ILC3 belonged to the desired effector subtype with high IL-22 and low IL-17 production. The protocol thus combines the advantages of avoiding xenogeneic components, which were necessary in previous protocols, with a high propensity for generation of IL-22-producing ILC3. The present approach is suitable for the generation of large amounts of ILC3 in an all-human system, which could facilitate development of clinical strategies for ILC3-based therapy in inflammatory diseases and cancer.

## Introduction

Recently, the therapeutic potential and possibility of translational approaches of human innate lymphoid cells (ILCs) has been highlighted by us (1) and others (2). One subset of ILCs is particularly interesting in this regard: tissue-resident ILC3. ILC3 have been described to express the transcription factor (TF) RORγT and can be divided into two functional subsets: NKp44^-^ILC3 secreting IL-17A and NKp44^+^ILC3 secreting IL-22 (3, 4). The latter have been recognized to be essential for promoting tissue integrity, maintaining barrier functions, and promoting homeostasis (3, 5), especially within the gastrointestinal (GI) tract. Secretion of IL-22 seems to play a key role for these functional properties (3). IL-22 secretion by NKp44^+^ILC3 is activated after food intake (6, 7), sensing danger signals (8, 9) as well as changes in cytokine milieu (3, 10). Furthermore, NKp44^+^ILC3s express neuroregulatory receptors enabling direct interactions with glial cells (11), but also interact with the microbiome (12) and goblet cells (13). The secreted IL-22 acts upon epithelial cells and intestinal stem cells (ISCs), both expressing the IL-22 receptor (IL-22R) (14), which is absent on cells from the hematopoietic lineage. Within experimental models, IL-22 stimulation had positive effects on ISCs to protect them against tissue damage (15–17) and promoted IFNλ secretion to induce anti-viral activity (18, 19). These studies combined suggest a complex interaction of IL-22-producing NKp44^+^ILC3 within the GI tract milieu necessary for our wellbeing.

Importantly, recent transplant studies have further strengthened the hypothesis that NKp44^+^ILC3s are essential for maintaining homeostasis and protection of the GI tract: following human GI transplantation, recipients` ILCs quickly infiltrated into the GI graft (20) and moreover elevated frequencies of human NKp44^+^ILC3s within the graft have been associated with successful intestinal transplants by reducing the risk of rejection (21). A similar observation has been made in leukemic patients after hematopoietic stem cell transplantation (HSCT). Patients undergoing allogeneic HSCT are in constant risk of graft-versus-host disease (GvHD) by alloreactive cells such as donor T cells attacking dominantly the skin, GI tract, and liver (22, 23). After HSCT, NKp44^+^ILC3, which are normally not present within the circulation of healthy individuals (24), occurred in peripheral blood and correlated with reduced risk of GvHD (25). The patient`s NKp44^+^ILC3s expressed chemokine receptors, potentially enabling the migration into the skin or GI tract and therefore possibly promoting protection. On the other hand, NKp44^-^ILC3 have been described to accumulate and to aggravate inflammation due to their secretion of IFNγ and IL-17A, respectively, especially during inflammatory bowel disease (IBD) (26–28). Therefore, IBD patients, leukemic patients undergoing HSCT, and GI-transplant recipients may benefit from cell-based therapies using human NKp44^+^ILC3.

Human effector ILC3s are predominantly found in the mucosa of certain organs, such as tonsils and intestine, and are virtually absent from the circulation. Of note, the circulating counterpart in umbilical cord blood (CB) referred to as ILC3-like cells are predominantly immature and lack typical ILC3 effector functions (29). Since the isolation of human ILC3 directly from organs appears not to be a viable option due to ethical issues and low cell recovery, the *in vitro* generation of ILC3 from HSPC appears to be a promising avenue. Indeed, in conditions enabling the generation of NK cell from HSPC *in vitro*, it has been already shown that besides NK cells, an additional population of IL-22-producing ILCs is generated (30, 31). The efficiency of ILC generation was increased by usage of murine stroma cells as stem cell niche (30, 32, 33). In this regard, we recently established a protocol for the generation of NK cells from CD34^+^ HSPCs in which murine stroma cells were replaced by human mesenchymal stem cells (MSCs) from bone marrow. The MSC-based niche enabled the highly efficient generation of NK cells including expression of KIRs, which are only scarcely generated on murine feeder cell lines (34). In the course of further studies analyzing the effect of human cytomegalovirus infection on NK cell differentiation *in vitro*, a NKp44^+^CD56^+^CD94^-^ cell subset with similarity to tissue ILC3s was identified by us (35). In the present study, we now provide compelling evidence that these cells indeed represent *bona fide* effector IL-22-secreting NKp44^+^ILC3s and that they can be efficiently generated from umbilical CB-derived HSPC in a human MSC niche. The *in vitro* generated NKp44^+^RorγT^high^ILC3 closely resemble tissue-resident ILC3 phenotypically and by global transcriptional analysis. Functionally, the *in vitro* generated NKp44^+^RorγT^high^ILC3 secreted high amounts of IL-22 and hardly any IL-17A. The present approach omits xenogeneic components and opens novel avenues for the efficient generation of IL-22-producing ILC3 in GMP-compatible conditions for future human cell-based therapies. 

## Results

### Efficient and Robust Generation of ILC3 from HSPC on Human MSCs

**Figure 1:**
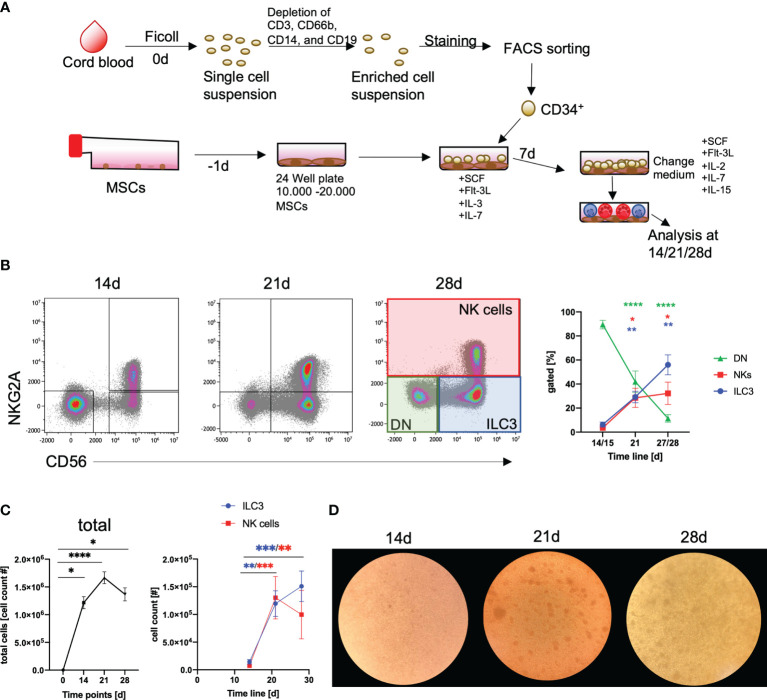
Efficient generation of ILC3 using an MSC-based differentiation platform. CD34^+^ HSPCs were isolated from fresh cord blood (CB) by cell sorting following depletion of unwanted cell populations (anti-CD3 for T cells, anti-CD66b for granulocytes, anti-CD14 for monocytes, and anti-CD19 for B cells). CD34^+^ HSPCs (2000/well) were seeded in 24-well plates with MSC, which were plated one day in advance **(A)**. Representative dot plots are shown for NKG2A and CD56 expression and frequency dynamics are shown for DN cells (NKG2A^-^CD56^-^, green box and line), ILC3 (NKG2A^-^CD56^+^, blue box and line), and NK cells (NKG2A^+^CD56^+^, red box and line), for day 0, 14, and 21 (*n* = 9), and day 28 (*n* = 7) (general gating strategy is shown in **Supplementary Figure 1**) **(B)**. Total cell counts (graph, left-hand side) as well as ILC3 (blue line) and NK cell (red line, both in graph, right-hand side) counts are shown for day 0, 14, and 21 (*n* = 9), and day 28 (*n* = 7) **(C)**. Representative microscopic pictures of cultures at day 14, 21, and 28 **(D)**. Error bars represent the mean ± SEM. The data are based on four different experiments with seven to nine individual CB donors and three different MSC lines. Levels of significance were calculated with a nonparametric ANOVA (Kruskal–Wallis with a Dunn`s post-test comparing day 14 to either day 21 or 28) **(B, C)**, *p < 0.05, **p < 0.01, ***p < 0.001, ****p < 0.0001.

The purpose of this study was to establish a standardized protocol for the GMP-compatible generation of human NKp44^+^ILC3 employing the HSPC/MSC platform, previously developed for the generation of NK cells (34). As outlined in **Figure 1A**, following isolation of mononuclear cells (MNCs) from CB, CD34^+^ HSPCs were sorted by flow cytometry, seeded on human MSCs, and differentiated for up to 28 days. Based on our previous study, NK cells and ILC3 could be distinguished on the basis of NKG2A and CD56 expression (35) (see **Supplementary Figure 1** for gating strategy). Thus, three major populations were provisionally identified: ILC3 (NKG2A^-^CD56^+^), NK cells (NKG2A^+^CD56^+^), and double negative (DN) cells (NKG2A^-^CD56^-^) (**Figure 1B**). In terms of cell frequencies, the three populations developed with distinct dynamics between day 14 and day 28: ILC3 frequencies continuously increased over time (ILC3: 14/15 days to 21 days, *p* = 0.001, 14/15 days to 27/28 days, *p* = 0.003), as did NK cells, but the latter with a slightly slower increase in the fourth week of culture (NK cells: 14/15 days to 21 days, *p* = 0.015, 14/15 days to 27/28 days, *p* = 0.029), while DN frequencies continuously decreased (14/15 days to 21 days, *p* < 0.0001; 14/15 days to 27/28 days, *p* < 0.0001) (**Figure 1B**). Thus, the culture conditions enabled a significant expansion of the different innate lymphoid subsets. Total cell counts strongly increased from 2 × 10^3^ CD34^+^ HSPC at day 0 to >1 × 10^6^ cells/well until day 21 (*p* < 0.0001). As expected, this was due to the expansion of ILC3 as well as NK cells (**Figure 1C**), while DN cells rather seem to be precursors due to their significant decline from day 14 to 28 and strong CD117 expression (data not shown). In the fourth week of culture, the ILC3 population further increased, whereas NK cell counts slightly dropped. Morphologically, clustering of typical comma-shaped cells, indicating the presence of NK cells and/or ILC3, was microscopically clearly apparent from day 21 of culture (**Figure 1D**). Of note, we used three different MSC lines to validate the stability of our differentiation platform and could not detect systematic differences in the generation of ILC3 between MSC lines, similar to previous observations looking at NK cell differentiation (34). All in all, the MSC-based differentiation platform proved to be a robust system for the *in vitro* generation of human ILC3.

### *In Vitro* Generated ILC3 and NK Cells Can Be Distinguished by Phenotype and Transcriptome

**Figure 2:**
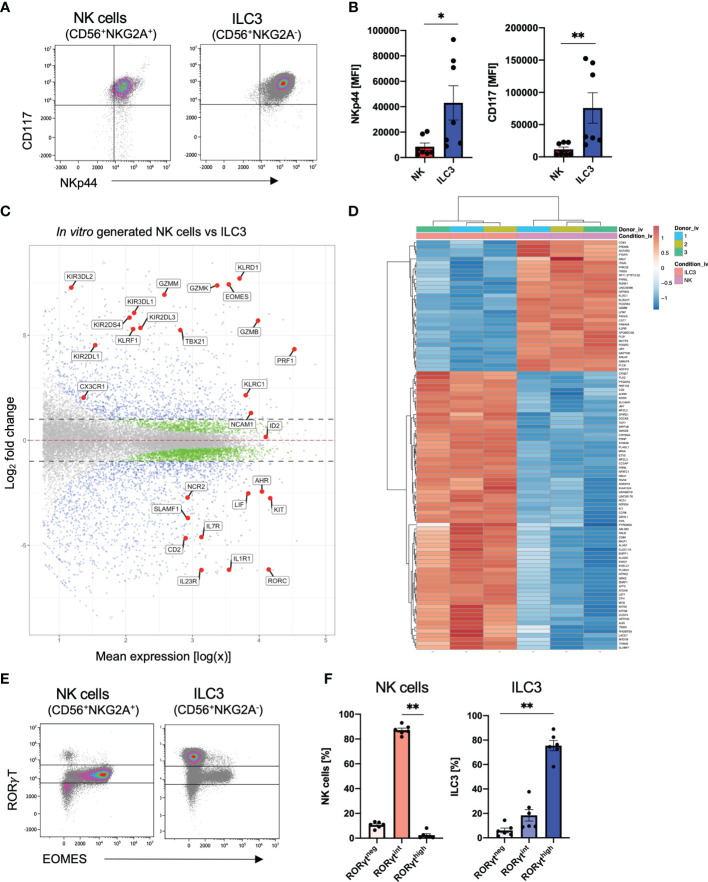
Distinct transcriptional profiles of *in vitro* generated ILC3 and NK cells. Freshly sorted CD34^+^ HSCs were seeded on MSC. On day 28, cultured cells were flow cytometrically analyzed **(A, B, E, F)** and NKG2A^+^CD56^+^ NK cells as well as NKG2A^-^CD56^+^ ILC3 were sorted for bulk RNA-seq analyses (*n* = 3 each). RNA sequencing was done on the Illumina platform **(C, D)**. Representative dot plots for CD117 and NKp44 expression of ILC3 and NK cells on day 28 **(A)**. Quantification of NKp44 (left-hand side) and CD117 (right-hand side) MFI for *in vitro* ILC3 and NK cells, *n* = 7 **(B)**. MA plot showing all differentially expressed genes between *in vitro* ILC3 and NK cells with dotted lines representing a log_2_ fold change cutoff of 1. Blue dots represent a *p*-value <.05 and a log_2_ fold change of >1. Green dots represent a *p*-value of <.05 and a log_2_ fold change of <1. Gray dots represent a *p*-value > 0.05 and a log_2_ fold change of <1. Selected blue genes are highlighted **(C)**. Heatmap showing the top 100 most differentially expressed genes between *in vitro* ILC3 and NK cells **(D)**. Representative dot plots of intranuclear EOMES and RORγT expression of NK cells and ILC3 **(E)**. Frequency distribution of NK cells and ILC3 within the RORγT^high^, RORγT^int^, and RORγT^neg^ fraction **(F)**. The data are representative of at least three different experiments **(A, B, E, F)**, and each dot is representative of an individual donor **(B, F)**. The height of the bar graphs represents the mean ± SEM. Level of significance was calculated with an unpaired nonparametric two-tailed *t*-test (Mann–Whitney test) **(B)** and a nonparametric ANOVA (Kruskal–Wallis with a Friedmann post-test) **(F)**, *p < 0.05, **p < 0.01.

Overall, ILC3 and NK cells follow quite similar kinetics during *in vitro* differentiation with first occurrence of both cell types around day 14 and strong increase until day 21. Furthermore, it is so far not possible to create culture conditions to generate one subset without the other. Notably, both ILC3s and NK cells expressed NKp44 and CD117 on the cell surface (**Figure 2A**); however, *in vitro* ILC3s showed significantly higher mean fluorescence intensity (MFI) values for NKp44 as well as CD117 (**Figure 2B**). To determine if our *in vitro* generated ILC3 and NK cells share transcriptional similarity to their *ex vivo* counterpart, we performed RNAseq analysis of bulk sorted ILC3 (sorted on NKG2A^-^CD56^+^) and NK cells (sorted on NKG2A^+^CD56^+^) to screen for distinguishing features. As outlined in **Figure 2C**, the transcriptomes of NK cells could be clearly distinguished from ILC3 based on the expression of typical NK cell receptors such as *KLRC1* (NKG2A), *KLRD1* (CD94), several killer cell immunoglobin-like receptors (*KIR2DL1, KIR3DL1, KIR3DL2*, and *KIR3DL2*), and *KLRF1* (NKp80) but also genes encoding granzymes (*GZMM, GZMK*, and *GZMB*) and perforin (*PRF1*) and essential TFs such as *TBX21* (TBET) and *EOMES* (**Figure 2C**). In contrast, *in vitro* ILC3 expressed hallmark genes of tissue-resident ILC3 such as *NCR2* (NKp44), *IL7R* encoding the ILC inclusion “marker” CD127, *CD2*, *KIT* (CD117), *IL1R*, and *IL23R* needed for cytokine stimulation of ILC3, as well as the ILC3 signature TFs *RORC* and *AHR* (**Figure 2C**). The distinct transcriptional signatures of *in vitro* generated ILC3 and NK cells are further clearly illustrated by a heatmap showing the top 100 differentially expressed genes. Notably, these data also illustrate how homogeneous the transcriptomic signatures of the *in vitro* generated ILC3s and NK cell populations are across different donors (**Figure 2D**).

We next looked more closely at previously defined signature TFs of ILC3 (RorγT) and NK cells (EOMES) at the protein levels. With regard to RorγT and EOMES, expression was validated by intranuclear staining: unexpectedly, we detected three different populations based on their RorγT expression: high (RorγT^high^), intermediate (RorγT^int^), and no (RorγT^neg^) expression (**Figure 2E** and **Supplementary Figure 2A**). NK cells were mainly RorγT^int^ with only a small fraction of RorγT^neg^ NK cells of approximately 10% (**Figure 2F**). Importantly, EOMES expression was almost completely restricted to the RorγT^int^ population, indicating that the CD56^+^NKG2A^+^ RorγT^int^EOMES^+^ population represents *bona fide* NK cells showing that NK cells also express RorγT but with lower expression levels compared to ILC3. In contrast, as expected, the RorγT^high^ population was lacking EOMES expression leading to a provisional phenotypic definition of *in vitro* ILC3 as CD56^+^NKG2A^-^RorγT^high^EOMES^-^. By gating on RorγT^high^EOMES^-^ and RorγT^int^EOMES^+^ gates, respectively, ILC3 and NK cells could be each defined with high confidence (**Supplementary Figure 2**). Notably, a small ILC3 population of unknown significance was co-expressing RorγT^int^ and EOMES (**Figures 2E, F**).

### The Transcriptome of *In Vitro* Generated ILC3 Is Closely Related to Tonsillar ILC3

**Figure 3:**
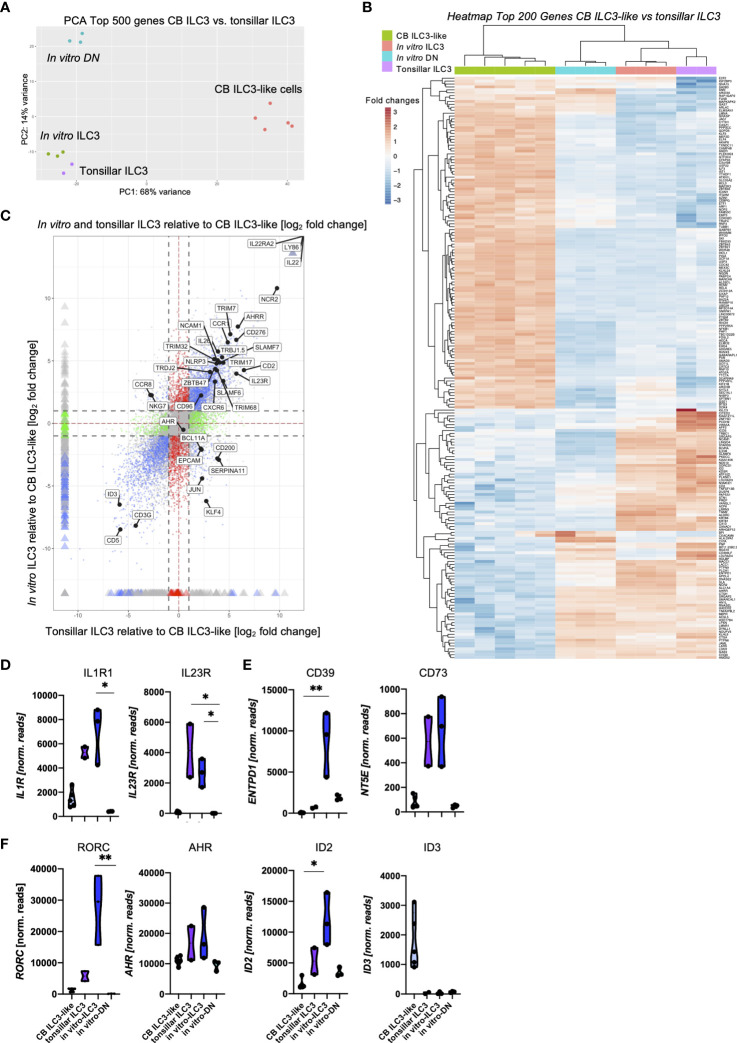
Shared transcriptomic signatures of *in vitro* generated ILC3 and tonsillar ILC3. Freshly sorted CD34^+^ HSCs were seeded on MSC and NKG2A^-^CD56^-^ DN cells and NKG2A^-^CD56^+^ ILC3 were sorted at day 14 and 28, respectively, for bulk RNA-seq analyses (*n* = 3 each). CB ILC3-like cells and tonsillar ILC3 were taken from a published dataset (29) (*n* = 5 for CB ILC3-like cells, *n* = 2 for tonsillar ILC3). RNA sequencing was done on the Illumina platform. A two-dimensional principal component analysis based on the top 500 differentially expressed genes of CB ILC3-like cells versus tonsillar ILC3 including the data of *in vitro* generated ILC3 and DN cells is shown **(A)**. A heatmap showing the top 200 differentially expressed genes between CB ILC3-like cells and tonsillar ILC3 with inclusion of *in vitro* ILC3 and DN cells **(B)**. A four-way plot with a cutoff at a log_2_ fold change ±1 (dotted lines) and adjusted *p*-values of.05 showing differently expressed genes of CB ILC3-like cells (“control”) compared to tonsillar ILC3 (“x”) and *in vitro* ILC3 (“y”). Blue dots represent genes with an adjusted *p*-value <.05 with a fold change >1. Green dots represent genes with an adjusted *p*-value <.05 with a fold change between >1 (*x*-axis) and <1 (*y*-axis). Gray dots represent genes with an adjusted *p*-value > 0.05. Red dots represent genes with an adjusted *p*-value <.05 with fold rates <1 (*x*-axis) and >1 (*y*-axis). Selected differentially expressed genes including ILC3 hallmark genes are highlighted **(C)**. Violin plots of normalized RNAseq read counts of known ILC3 functional receptors *IL1R* and *IL23R* **(D)**, ectoenzyme expression *ENTPD1* and *NT5E* **(E)**, and transcription factors *RORC, AHR, ID2*, and *ID3* **(F)** for CB ILC3-like cells (light blue), tonsillar ILC3 (purple), *in vitro* ILC3 (royal blue), and DN cells (aqua). Level of significance was calculated with a nonparametric ANOVA (Kruskal–Wallis with a Dunn`s post-test) **(B, C)**, *p < 0.05, **p < 0.01.

A key question that we wanted to address in this study pertains to the similarity between the *in vitro* generated ILC3 and *ex vivo* tissue-resident ILC3 in terms of phenotype, transcription, and function. As a first step, we compared *in vitro* generated ILC3 with tonsillar ILC3, representing a well-defined archetypical ILC3 effector cell subset, by bulk RNA-seq analyses. To be most unbiased, we performed a two-dimensional principal component analyses (PCA) based on the top 500 most differentially expressed genes between the *ex vivo* subsets of tonsillar ILC3 and CB ILC3-like cells [published dataset (29)] compared to the new data of the *in vitro* generated ILC3 as well as the DN (NKG2A^-^CD56^-^) population. We have recently characterized CB ILC3-like cells in depth and observed that although this subset seems to be phenotypically similar to tissue-resident ILC3, they largely lack typical ILC3 effector function and have a different transcriptomic signature (29). As shown in **Figure 3A**, *in vitro* generated ILC3 closely clustered to tonsillar ILC3, whereas CB ILC3-like cells were located far apart within the first principal component (PC1) representing 68% of the variance. *In vitro* generated DN cells were similar in the PC1 but clearly separated in PC2, representing 16% of variance. We next performed unsupervised clustering of the top 200 most differentially expressed genes between CB ILC3-like cells and tonsillar ILC3 (**Figure 3B**). The resulting heatmap again demonstrated a close similarity between tonsillar and *in vitro*-generated ILC3, clustering apart from CB ILC3-like cells as expected but also showing several divergent gene clusters in comparison to the DN population. Finally, to further test the transcriptional similarity and determine shared gene signatures of *in vitro* ILC3 and tonsillar ILC3, a four-way plot with CB ILC3-like cells as reference sample was calculated. The four-way plot identifies a large number of genes commonly expressed in tonsillar ILC3 that are also expressed in *in vitro* generated ILC3 (upper right quadrant), further validating that *in vitro* generated ILC3 are very similar to *ex vivo* tonsillar ILC3s (**Figure 3C**). Among the shared genes are many, which have been described to be hallmarks for tissue-resident ILC3, such as *IL23R, IL22*, and *NCAM1* encoding CD56, *NCR2* encoding NKp44, and *AHRR* encoding AHR repressor, as well as many other shared genes. Nonetheless, some unique genes distinguishing *in vitro* generated ILC3 and tonsillar ILC3 were identified such as *CCR8* and *NKG7* for the *in vitro* generated ILC3s (upper left quadrant) and *CD200*, *KLF4*, and *EPCAM* for tonsillar ILC3 (lower right quadrant). Finally, the four-way plot also identified a unique set of genes known to be expressed in CB ILC3-like cells, such as the TF *ID3* (29).

We next looked at the expression levels of selected ILC3-specific genes (**Figures 3D–F**). Tonsillar and *in vitro* generated ILC3 had comparable read counts for *IL1R1* and *IL23R* encoding the receptors stimulated by IL-1β and IL-23, constituting the presumably most important stimuli for ILC3 function (3) (**Figure 3D**). Whereas expression of IL23R could not be validated on the cell surface due to lack of suitable staining reagents, *in vitro* ILC3 revealed strong expression of the IL1R1 expression (**Supplementary Figure 3**). Both receptors were either low (IL1R1) or negative (IL23R) in CB ILC3-like and also completely negative in DN cells. Recently, a novel ectoenzyme-expressing subset of ILC3 (ecto^+^ILC3) has been identified within the oral-GI tract and bone marrow (BM) that is reactive to extracellular adenosine triphosphate (eATP), which upon activation have shown immunosuppressive capacities against autologous T cells (8). Given its potential relevance for ILC3-based therapy of GvHD, we checked the respective ectoenzymes *NT5E* encoding CD73 and *ENTPD1* encoding CD39. Indeed, *NT5E* was strongly expressed in tonsillar as well as *in vitro* ILC3, whereas it was very low in CB ILC3-like and *in vitro* DN cells. Flow cytometrically analyses revealed that most *in vitro* ILC3 expressed CD39, but only a fraction expressed CD73 (**Supplementary Figure 3**). Interestingly, the ectoenzyme *ENTPD1*, which was shown to protect from intestinal injury in colitis (36), was found to be strongly expressed in *in vitro* ILC3 whereas it was weak in tonsillar ILC3, CB ILC3-like, and DN cells (**Figure 3E**).

*In vitro* ILC3 also exhibited high expression levels of ILC3-specific transcription factors: we observed a 4.8-fold higher expression for RORC compared to tonsillar ILC3 (**Figure 3F**) and, as previously reported, hardly any expression for CB ILC3-like cells (29). The aryl-hydrocarbon receptor *AHR* exhibited comparable expression within *in vitro* and tonsillar ILC3 with only slightly higher read counts compared to CB ILC3-like cells and *in vitro* DN cells. Finally, the inhibitor of DNA binding factors *ID2* and *ID3* have been described to play major roles within innate lymphoid lineage fate decisions (37, 38), and in particular, ID2 has been described to be vital for ILC development in general (39). We observed highest *ID2* expression in *in vitro* ILC3 with distinctly lower levels in tonsillar ILC3 and only marginal expression within CB ILC3-like cells and DN cells. In line with our previous observations (29), ID3 was strongly expressed in CB ILC3-like cells, whereas it was not expressed in other ILC populations (**Figure 3F**). Altogether, the transcriptomic signature of *in vitro* ILC3 was shown to be closely related to *ex vivo* tonsillar ILC3.

### *In Vitro* Generated ILC3 Provide a Superior Profile of Effector Functions Including High IL-22 Production and Lack of IL-17A

**Figure 4:**
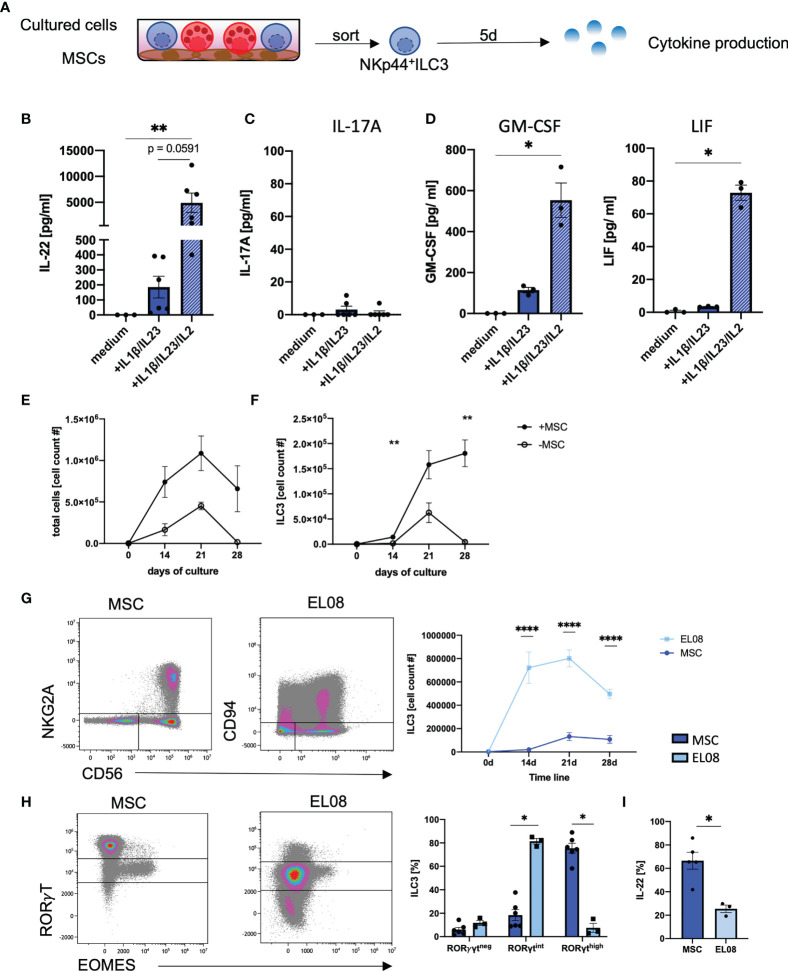
*In vitro* generated ILC3 produce high amounts of IL-22, but not IL17a. At day 28 on MSCs, NKp44^+^ILC3s were freshly sorted and stimulated either with medium alone or with IL1β/IL-23 (50 ng/ml each) with or without IL-2 (1000 U/ml). Supernatant was taken at day 5 **(A)**. IL-22 **(B)**, IL-17A **(C)**, GM-CSF, and LIF **(D)** levels in the supernatant were measured after a 5-day stimulation with medium alone, IL1β/IL-23 (blue bar), and IL1β/IL-23/IL-2 (blue-white striped bar). CD34^+^ HSPCs were sorted and 2,000 cells were seeded in parallel in wells with MSC (+MSC) or without MSC (-MSC). Line graphs showing total cell counts **(E)** and ILC3 (NKG2A^-^CD56^+^) cell counts **(F)** in cultures with MSC (+MSC, filled dots, *n* = 5–7) and without MSC (-MSC, open dots, *n* = 3) from day 0 to day 28. CD34^+^ HSPCs were cultured on EL08. Representative dot plots of NKG2A and CD56 expression (left panels) and quantification of ILC3 (right panel) cultured on EL08 (light blue, *n* = 3) or on MSC (dark, blue, *n* = 7–9) **(G)**. Representative dot plots showing RORγT and EOMES expression (left panels) and quantification of RORγT^neg^, RORγT^int^, and RORγT^high^ frequency distribution (right panels, bar graphs) for cells cultured on EL08 (light blue, *n* = 3) or on MSC (dark blue, *n* = 7) **(H)**. Cultures derived on MSC or EL08 were re-stimulated with IL1β/IL-23 and stained for intracellular IL-22 secretion. Bar graphs showing IL-22 secretion for MSC-derived ILC3 (dark blue, *n* = 5) and EL08-derived ILC3 (light blue, *n* =3). The data are representative of one (EL08 and culture -MSC) and two different experiments (+MSCs) with each dot representative of an individual donor. The height of the bar graphs represents the mean ± SEM. Levels of significance were calculated with a nonparametric ANOVA (Kruskal–Wallis with a Dunn`s post-test) **(B–D)** and Mann–Whitney tests **(E–I)**, *p < 0.05, **p < 0.01, ****p < 0.0001.

We next examined cytokine-based effector functions of *in vitro* ILC3. In this regard, the efficient production of IL-22 is highly relevant given its described beneficial function for tissue regeneration and repair within the GI tract (7, 15, 40). To this end, we flow cytometrically sorted *in vitro* generated NKp44^+^ILC3 and stimulated the cells with medium alone, IL1β/IL-23 (3), or IL1β/IL-23/IL-2 (10) (**Figure 4A**). Surprisingly, the classical ILC3 stimulation protocol using IL1β/IL-23 could be significantly improved by addition of IL-2, leading to substantially increased secretion of IL-22 (**Figure 4B**). ILC3 have also been described to produce IL-17A, which acts inflammatory when produced in larger quantities (41). However, hardly any IL-17A protein secretion was found with any stimulus we used (all means < 5 pg/ml) (**Figure 4C**), which is in line with the hypothesis that NKp44^+^ILC3 secrete IL-22 whereas NKp44^-^ILC3 secrete IL-17A (4). Furthermore, in line with published data of *ex vivo* tonsillar ILC3s (10), we detected significantly higher amounts of GM-CSF and LIF when adding IL-2 to the stimulation (**Figure 4D**) (10). Of note, stimulation did not change the overall ILC3 phenotype of CD56^+^NKG2A^-^CD117^+^ (data not shown). Thus, *in vitro* ILC3 are high producers of IL-22 without accompanying secretion of IL-17A.

We next wanted to compare the efficiency of MSC-based differentiation of *in vitro* ILC3 to previously established protocols (30–32, 42). We first compared our MSC-based protocol to a cytokine-only milieu using the identical media conditions. We observed a similar trend of cell proliferation during both culture conditions with an increase in cell numbers until day 21 with overall stronger proliferation in MSC-based conditions. However, a sharp decline accompanied by massive cell death (data not shown) was observed during the fourth week of culture in the cytokine-only conditions compared to a slight decline using the MSC-based approach (**Figure 4E**). Thus, while *in vitro* ILC3 constantly increased in cell number up to day 28, as already shown in **Figure 1**, a sharp decline was observed for ILC3 in cytokine-only conditions (**Figure 4F**). Hence, compared to the MSC-based approach, the cytokine-only conditions do not appear to be suitable for efficient expansion of ILC3.

Finally, the MSC-based approach was compared to a protocol, using the murine embryonic stroma cell line EL08, which was established by the Verneris group and is widely regarded as the gold standard for generation of ILC3 (30, 32). Indeed, when comparing both methods, we observed significantly higher frequencies of ILC3s on murine EL08 cells at day 14, 21, and 28 of culture (**Figure 4G**). However, when we compared the expression of RorγT, ILC3 generated on murine EL08 mainly exhibited a RorγT^int^ phenotype whereas ILC3 on human MSC largely exhibited a RorγT^high^ phenotype. Although we had shown above that the RorγT^int^ phenotype is largely restricted to NK cells in MSC-based cultures, this was not the case for EL08-supported cells, since they lacked expression of EOMES (**Figure 4H**). Finally, MSC-based generation of ILC3 led to significantly higher IL-22 secretion than ILC3 generated on EL08 (**Figure 4I**). Altogether, MSC-supported differentiation of CB-derived HSPC enables robust and efficient generation of human RorγT^high^ILC3 secreting high amounts of IL-22 in fully human culture conditions.

## Discussion

In this study, we present a detailed protocol for the efficient and GMP-compatible generation of human effector ILC3 from CB-derived hematopoietic progenitors. The utilization of human clinical-grade MSC for the support of hematopoietic differentiation into the ILC3 lineage enables, for the first time, an efficient xenobiotic-free way to generate IL-22-producing ILC3. The protocol is robust and works efficiently with different donors and MSC lines. The *in vitro* generated ILC3s were functionally, phenotypically, and transcriptionally comparable to tissue-resident ILC3s by expressing high levels of NKp44, CD117, RorγT, as well as secreting high amounts of IL-22, but not IL-17A. Transcriptomic analyses demonstrated a close similarity between tonsillar and *in vitro* generated ILC3, which was not necessarily expected, given the fact that this involved comparison of an *ex vivo* with an *in vitro*-generated cell subset. The close relationship between *in vitro* and tonsillar ILC3 was especially evident with respect to genes responsible for ILC3 effector functions, setting both cell types apart from circulating ILC3-like cells that are basically unable to produce IL-22 (24, 29). Of note, when relaxing the analysis parameters of the RNAseq data by accounting for all genes, the effect of cell culturing becomes dominant and *in vitro* ILC3 become more closely related to *in vitro* DN cells taken from the same culture dish compared to the tonsillar ILC3 (data not shown). Furthermore, the study shows that the generation of human ILC3s on MSCs leads to favorable results in terms of IL-22 production compared to previous protocols employing either murine stroma cells (30, 32, 33, 43) or feeder-free conditions (31, 44–46). Notably, bone-marrow-derived MSCs were previously described to promote proliferation and enhanced IL-22 secretion of *ex vivo* isolated tonsillar ILC3 (47), further suggesting that MSC besides providing an efficient stem cell niche, as shown here, are also capable of supporting their effector functions.

Importantly, ILC3 generated on MSC produced high amounts of IL-22 with no co-secretion of IL-17A. This is an essential observation, as IL-17A was previously shown to exacerbate inflammation in IBD, whereas IL-22-secreting ILC3s have been described to be beneficial for IBD patients, leukemic patients after HSCT, and GI tract transplant recipients (15, 21, 25, 27, 48–50). Several issues still have to be addressed concerning the role of ILC3 in inflammatory settings: an increase in IL-17A-producing ILC3s was reported in IBD, correlating with mucosal leakage in ulcerative colitis patients (27, 51). These observations could be due to a switch in effector programs from IL-22- to IL-17-producing ILC3, which would be undesirable for immunotherapy due to IL-17-mediated aggravation of inflammation. We did not experience a switch or a *de novo* expression of IL-17A during *in vitro* generation of ILC3 and also not following prolonged culture and challenge with different stimuli. Nonetheless, the point of a potential plasticity of the *in vitro* generated ILC3 needs to be addressed in the future, employing suitable *in vivo* and *in vitro* models.

The interest in developing protocols for ILC3-based cellular therapy was recently fueled by observations made in clinical GI transplantation. It has been observed that human ILCs quickly infiltrate the transplanted GI tract with >70% of ILCs being recipient-derived (20). This is in line with a recent murine study showing that IL-22-secreting mouse ILC3s were able to infiltrate into transplanted lungs promoting the formation of peripheral nodal addressin-expressing high endothelial venules (52). Importantly, the frequency of human NKp44^+^ILC3s correlated with successful engraftment of the intestinal transplants. Due to the high correlation and prediction success, human NKp44^+^ILC3s were suggested to be a biomarker for transplant rejections (21). Both studies combined highlight that human NKp44^+^ILC3s are beneficial for GI transplantation success, but also that NKp44^+^ILC3s are able to actively migrate into tissues. These studies provide a paradigm change to the previous notion that ILC3s are largely tissue resident and renew rather locally than by replenishment from external progenitors or migration of mature ILCs into the tissues (53). Hence, although this has to be proven in future studies, it is reasonable to believe that intravenously given NKp44^+^ILC3s would find their way into GI tissues. Thus, *in vitro* generated IL-22-producing NKp44^+^ILC3s could exert beneficial effects in the settings of GvHD following allogeneic HSCT and could also provide a promising new approach for treatment of intestinal inflammatory processes in the colon and small intestine of Colitis Ulcerosa and Morbus Crohn patients, respectively. Notably, there is so far no curative treatment available in either setting. The present approach for the efficient generation of clinically applicable ILC3 could facilitate first steps into this translational direction.

It has to be acknowledged that our *in vitro* cultures besides ILC3 predominantly contained NK cells. This is observed in all *in vitro* differentiation approaches reported so far and is probably due to the predominance of common progenitors for NK cells as well as ILC3 within the CD34^+^ HSPC fraction. On the other hand, the cultures were completely free of critical T-cell contaminations (data not shown) and none of the non-ILC3 cellular components within the culture is expected to constitute a safety issue for clinical application. It has to be stressed that previous experience with NK cell-based therapies did not point towards any GvHD-promoting effect of allogeneic NK cells (54). In fact, contaminating NK cells might rather have immunosuppressive effects due to regulation of inflammatory CD8^+^ T cells (55). Similarly, MSCs are endowed with immunosuppressive features (56) and any remaining MSC contamination in the culture would not constitute any hazard for the clinical product in contrast to murine feeder cells that would have to be completely removed before clinical use. In any case, it would be feasible to generate highly pure ILC3 products for example by applying a two-step protocol for GMP-compatible enrichment (i.e., magnetic beads) involving depletion of NKG2A^+^ NK cells and subsequent enrichment of NKp44^+^ILC3.

Classically, murine stroma cells are employed to support the differentiation of human HSC into mature immune cell types such as NK cells, T cells, and more recently ILCs (30, 32, 33, 57–63). This is based on the premise that the major interactions between hematopoietic stem cells and niche cells are conserved between mouse and humans. However, many important players, such as cytokine receptors, adhesion molecules, and chemokine receptors, are not compatible with the respective murine ligands and might in part explain the superior function of human MSC in this context. It would certainly merit further investigation to define how far these species-specific differences are modulating differentiation programs. Regarding conserved stroma-defined signals, we and others have shown that ectopic expression of human NOTCH ligands such as DLL1 and DLL4 into murine OP9 stroma cells enhanced the efficiency of differentiation into the NK cell lineage (34, 59, 63) and, as recently shown, similarly into the ILC lineage (33). Notably, DLL proteins do not seem to play a major role for MSC-based differentiation of ILC3, since the MSC lines consistently showed either no (DLL4) or only very low (DLL1 and DLL2) expression in RNAseq analyses (data not shown). In fact, Jagged 1 represented the only highly expressed NOTCH ligand in MSC, but its role in this system needs to be explored in the future. In general, it is well established that MSCs constitute one of the key components of the bone marrow stem cell niche (64), and its usage in the present protocol will facilitate future studies of ILC3 development without species-specific receptor/ligand incompatibilities found in xenogeneic settings, mirroring more closely *in vivo* development.

Another advantage of the present approach for generation of human effector ILC3 is the lack of regulatory hurdles attached to MSC in contrast to the use of murine feeder cells. In the latter case, master cell banks have to be established and stringent proof must be provided excluding the risk for transmission of zoonotic pathogens, which precludes clinical phase III studies in many countries. In contrast, human MSCs are used within clinical trials in various disease settings [reviewed here (65)]. The MSC lines used within this study were established from BM of children under GMP conditions and have been subjected to GMP quality controls as described previously (34). These MSC lines are routinely used in the clinic for treatment of GvHD, and thus fully meet the regulatory criteria for the use of clinical products (66). Even though the success of MSC-based therapies is controversial (56), the fact that MSCs are already used in clinical trials shows that it is ethically and regulatory unproblematic to be used within humans. In practical terms, human MSC lines have a high renewal capacity and can be expanded to large quantities if needed. In our experience, the *in vitro* generation of ILC3 as well as NK cells (34) did not require the usage of particularly early MSC passages but worked equally well with more advanced MSC lines, which facilitated the acquisition of consistent results over time. Adding to this, the present experiments were conducted using three different donor-derived MSC lines, and in fact, all three MSC lines supported a robust generation of human NKp44^+^ILC3s enabling narrow error margins. Altogether, our protocol enables the robust and GMP-compatible generation of fully functional human IL-22-producing ILC3, hopefully paving the way for their future therapeutic application.

## Materials and Methods

### Human Samples and Ethics Statement

Umbilical CB used within this study was collected by the José Carreras Stem Cell Bank at the ITZ. The protocol was accepted by the institutional review board at the University of Düsseldorf (study number 2019-383) and is in accordance to the Declaration of Helsinki. CBs were processed directly without previous thawing.

### Cell Culture

GMP-grade MSCs were obtained as previously described (34). For the experiments, three different MSC lines were used. MSCs were cultured in Dulbecco’s high-grade glucose medium (Gibco) with 10% platelet lysate and 1% penicillin/streptomycin (Stock: 10,000 U/ml penicillin; 10,000 μg/ml streptomycin, Gibco). MSCs (10,000–20,000) were plated 1–2 days before the CD34^+^ isolation in the inner eight wells of a 24-well plate (Gibco Tissue-treated cell culture plates). The murine embryonic liver stroma cell line EL08-1D2 (= EL08) was cultured in Iscove modified Dulbecco medium (Lonza) with 15% fetal calf serum, 5% horse serum, 1% penicillin/streptomycin, and 400 mM 2-mercaptoethanol at 33°C (67, 68). For generation of ILC3, 2,000 freshly sorted CD34^+^ hematopoietic stem cells (day 0) were seeded on the MSCs, EL08, or without feeder cells in “NK1” medium for 7 days. On day 7, half the NK1 medium was removed and a new “NK2” medium was added. “NK1” and “NK2” medium had been previously described by us (35) except that the “NK1” medium did not contain IL-2 in the present protocol. Half of the medium was changed twice a week. Flow cytometric analyses were done at days 14/15, 21, and 27/28 of culture. The cells are harvested from the wells by careful pipetting and ensuring by visible inspection that all cells have been detached from the well.

### Isolation of MNCs From Cord Blood and Sorting of CD34^+^ Cells

CB was diluted 1:1 with sterile 1×PBS (Gibco by Life Technologies, California, USA) and MNCs were isolated *via* a density gradient centrifugation (Biocoll, 1.077 g/cm^3^, Biochrom Merck). For erythrocyte lysis, cells were resuspended in 5 ml of ice-cold ammonium chloride solution (pH = 7.4, University Clinic Düsseldorf) with three consecutive washing steps. MNCs were counted and further prepared for sorting. Monocytes, B cells, and T cells were depleted *via* MojoSort Streptavidin Nanobeads (BioLegend) using the supplier’s negative selection protocol as previously described (63). The cells were stained with a lineage panel as previously described (69), as well as CD94, CD127, CD117, and CD34 for sorting of CD34^+^ HPCs. Cell sorting was performed on a MoFlo XDP (Beckman Coulter).

### Flow Cytometric Analyses

Cells were extracellularly stained with the following FITC-conjugated lineage antibodies for sorting as previously described (69): anti-CD3 (UCHT1), anti-CD1a (HI149), anti-CD14 (HCD14), anti-CD19 (HIB19), anti-TCRαβ (IP26), anti-TCRγδ (B1), anti-CD123 (6H6), anti-CD303/BDCA-2 (201A), anti-FceR1a [AER-37(CRA-1)], anti-CD235a (HI264), and anti-CD66b (G10F5), all from BioLegend. These antibodies were further used within the study: anti-CD3-APC/Cy7 (UCHT1), anti-CD14-APC/Cy7 (HCD14), anti-CD19-APC/Cy7 (HIB19), anti-CD56-PE/Dazzle™ 594 or BV650™ (HCD56), anti-CD34-BV510™/-PE or -FITC (581), anti-NKp44-APC (P44-8), anti-CD73-PE/Dazzle™ 594 (AD2), anti-CD39-APC (A1), anti-Thy1-Alexa Flour^®^ 700 (5E10), and anti-CD117-BV421 and -PE/Cy7 (104D2), all from BioLegend. CD127-PE/Cy5 (R34.34) and NKG2A-PE/Cy7 (Z199) were from Beckman Coulter (California, USA). Anti-IL1R1-PE (FAB269P) was purchased from R&D (Minneapolis, Minnesota). Intranuclear staining of anti-Eomes-PE-eFlour^®^ 610 (WD1928, Invitrogen) and anti- RorγT-PE (clone: AFKJS-9, eBioscience) was performed with the Foxp3 staining kit (Thermo Fischer Scientific) and protocol. The initial gating strategy is shown in **Supplementary Figure 1**.

### Functional Analyses

For functional analyses, cells were stimulated with human (h) IL-1β (50 ng/ml) and IL-23 (50 ng/ml) with or without IL-2 (1,000 U/ml) overnight with subsequent addition of Brefeldin A solution (1,000×, BioLegend, 1,000-fold dilution) after 1 h of stimulation. The cells were stained extracellularly for detection of ILC3 and NK cells as well as intracellularly for analysis of IL-22 (2G12A41, BioLegend) production using the intracellular staining kit (BioLegend). *In vitro* generated ILC3 were flow cytometrically sorted (see RNA sequencing and analyses for gating) and 50,000 cells were subsequently stimulated for 5 days. Supernatant was collected at day 5. LEGENDplex Human Th Panel and the LEGENDplex Human Hematopoietic Stem Cell Panel from LEGENDplex (Cat: 740722 and 740611, BioLegend, San Diego, California) were used and experiments were done according to the manufacturer’s instructions.

### RNA Sequencing and Analyses

On day 28, cells were taken from cultures, stained, sorted for ILC3 (Lin^-^CD34^-^Thy1^-^NKG2A^-^CD56^+^NKp44^+^) and NK cells (Lin^-^CD34^-^Thy1^-^NKG2A^+^CD56^+^), and stored in TRIzol™ Reagent (Invitrogen) for extraction of total RNA. Reverse transcription and library production were carried out with an Illumina Truseq RNA preparation kit as described in the company’s protocol. Sequencing of the libraries was performed with an Illumina HiSeq4000 (single-read 1×50bp). Sequence reads were mapped to the human genome (hg38) with STAR (version STAR_2.5Oa), and read counts of gene transcripts were determined using gtf file Homo_sapiens.GRCH38.84.gtf and featureCount (v1.5.0-p1). Analysis of differential gene transcription and normalization of read counts and PCA were performed with R package DESeq2 (v.1.22.2) (70), and four-way plots and MA plots were generated with R package vidger (v.1.2.1) (71).

### Statistical Analyses

All tests were performed with a nonparametric assumption and significance level of 0.05. All analyses were done using GraphPad Prism 8.0.0 (GraphPad Software, San Diego, California, www.graphpad.com).

## Data Availability Statement

RNA sequencing data for ILC3-like cells and in vitro generated ILC3 and NK cells is accessible at NCBI Project ID: PRJNA642003 (https://www.ncbi.nlm.nih.gov/bioproject/?term=PRJNA642003) and PRJNA777910 (https://www.ncbi.nlm.nih.gov/sra/PRJNA777910).

## Ethics Statement

The studies involving human participants were reviewed and approved by Ethikkommission, Medical Faculty, University hospital Düsseldorf (study number 2019-383). Written informed consent to participate in this study was provided by the participants’ legal guardian/next of kin.

## Author Contributions

SB: Conception and design, collection and/or assembly of data, data analysis and interpretation, manuscript writing, and final approval of the manuscript. SW: Collection and/or assembly of data, conception and design, and final approval of the manuscript. ÖD: Collection and/or assembly of data, provision of study material, and final approval of the manuscript. RO: Provision of study material and final approval of the manuscript. KR: Collection and/or assembly of data and final approval of the manuscript. GK: Provision of study material, conception and design, and final approval of the manuscript. RM: Collection and/or assembly of data, provision of study material, and final approval of the manuscript. LW: Collection and/or assembly of data, manuscript writing, and final approval of the manuscript. MU: Conception and design, data analysis and interpretation, manuscript writing, financial support, and final approval of the manuscript. All authors contributed to the article and approved the submitted version.

## Funding

This work was supported by funds from the Düsseldorf School of Oncology (funded by the Comprehensive Cancer Center Düsseldorf/Deutsche Krebshilfe and the Medical Faculty HHU Düsseldorf) and the Deutsche Forschungsgemeinschaft (DFG) FOR2033, TP B3 (RO), SPP1937-UH91/8-1 and UH91/10-1 (both MU). This work was partially supported by funds from the Bundesministerium für Bildung und Forschung (BMBF) MyPred, 01GM1911E to RM.

## Conflict of Interest

The authors declare that the research was conducted in the absence of any commercial or financial relationships that could be construed as a potential conflict of interest.

## Publisher’s Note

All claims expressed in this article are solely those of the authors and do not necessarily represent those of their affiliated organizations, or those of the publisher, the editors and the reviewers. Any product that may be evaluated in this article, or claim that may be made by its manufacturer, is not guaranteed or endorsed by the publisher.
