# Corrigendum: Efficient *in vitro* generation of IL-22-secreting ILC3 from CD34+ hematopoietic progenitors in a human mesenchymal stem cell niche

**DOI:** 10.3389/fimmu.2023.1292209

**Published:** 2023-09-22

**Authors:** Sabrina B. Bennstein, Sandra Weinhold, Özer Degistirici, Robert A. J. Oostendorp, Katharina Raba, Gesine Kögler, Roland Meisel, Lutz Walter, Markus Uhrberg

**Affiliations:** ^1^ Institute for Transplantation Diagnostics and Cell Therapeutics, Medical Faculty, Heinrich-Heine University Düsseldorf, Düsseldorf, Germany; ^2^ Division of Pediatric Stem Cell Therapy, Clinic for Pediatric Oncology, Hematology and Clinical Immunology, Center for Children and Adolescence Health, Heinrich-Heine University Düsseldorf, Medical Faculty, Düsseldorf, Germany; ^3^ Technical University of Munich, School of Medicine, Klinikum rechts der Isar, Department of Internal Medicine III – Hematology and Oncology, Laboratory of Stem Cell Physiology, Munich, Germany; ^4^ Primate Genetics Laboratory, German Primate Center, Leibniz Institute for Primate Research, Göttingen, Germany

**Keywords:** CD34 cells^+^, mesenchymal stem cells, umbilical cord stem cells (UCSC), innate lymphoid cells (ILCs), ILC3, hematopoietic stem and progenitor cells, GMP—Good Manufacturing Practice

In the published article, there was an error in the Funding statement. A funding source of the author Roland Meisel was mistakenly not included in the publication. The correct Funding statement appears below.

## Funding

This work was supported by funds from the Düsseldorf School of Oncology (funded by the Comprehensive Cancer Center Düsseldorf/Deutsche Krebshilfe and the Medical Faculty HHU Düsseldorf) and the Deutsche Forschungsgemeinschaft (DFG) FOR2033, TP B3 (RO), SPP1937-UH91/8-1 and UH91/10-1 (both MU). This work was partially supported by funds from the Bundesministerium für Bildung und Forschung (BMBF) MyPred, 01GM1911E to RM.

The authors apologize for this error and state that this does not change the scientific conclusions of the article in any way. The original article has been updated.

