# Supplementary material for: Efficient In Vitro Generation of IL-22-Secreting ILC3 From CD34+ Hematopoietic Progenitors in a Human Mesenchymal Stem Cell Niche
Source: Front Immunol. 2021 Dec 24;12:797432. doi: 10.3389/fimmu.2021.797432 (PMC8739490; doi:10.3389/fimmu.2021.797432)
Supplement: Supplementary file 1 [file DataSheet_1.pdf]

Supplementary Material

1.1 Supplementary Figures

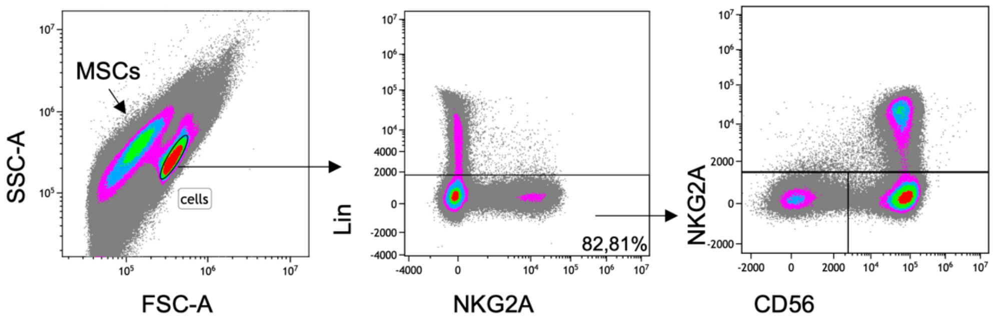

Supplementary Figure 1 | Gating strategy for *in vitro* culture Representative gating strategy for *in vitro* ILC3 and NK cell identification on day 28 of culture. Generated cells can be distinguished from MSCs via Forward Scatter area (FSC-A) and Side Scatter Area (SSC-A) (left hand side). Lineage (Lin) positive cells (comprising monoclonal antibodies against CD3, CD19, and CD14) are excluded (middle). Lin^- cells are further gated on CD56 and NKG2A to distinguish ILC3 ($CD56^+NKG2A^-$ cells), NK cells ($CD56^+NKG2A^+$), and double negative cells ($CD56^-NKG2A^-$ cells) (right hand side).

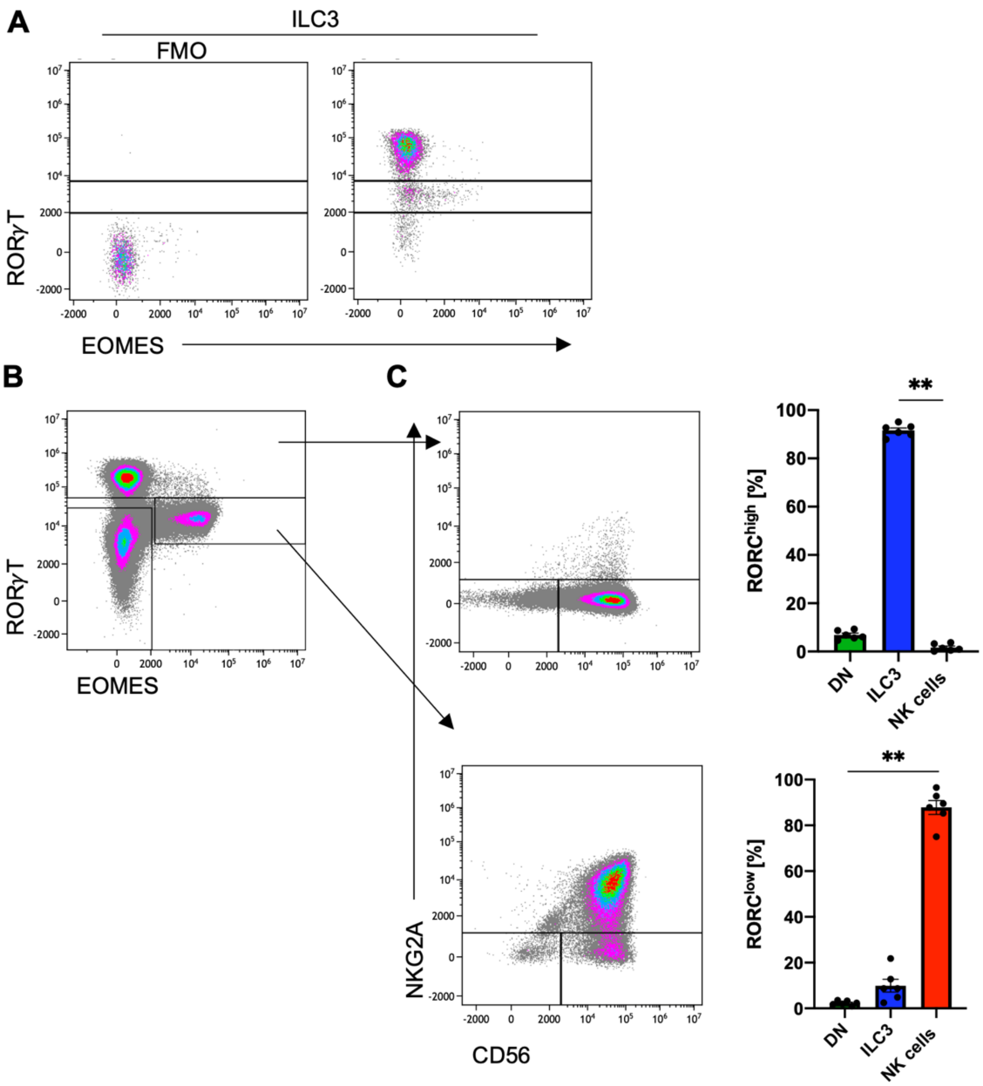

Supplementary Figure 2 | Identification of *in vitro* ILC3 and NK cells based on RORγT expression levels. Representative dot plot of RORγT and EOMES comparing FMO control (left hand side) with normal staining (right hand side) gated on ILC3 (A). Representative dot plot of intranuclear staining for RORγT and EOMES expression gated on Lin$^-$ cells (B). Representative dot plots of NKG2A and CD56 showing the distribution and quantification of the RORγT^{high} (top) and RORγT^{int} (bottom) population with respect to DN cells (NKG2A$^-$CD56$^-$), ILC3 (NKG2A$^-$CD56$^+$), and NK cells (NKG2A$^+$CD56$^+$), n=6 (B). The data is representative of at least three individual experiments (B/C) or one individual experiment (A) with each dot representing an individual donor. The heights of the bar graphs represent the mean ± SEM. Level of significance were calculated with a nonparametric ANOVA (Kruskall-Wallis with a Dunn`s post-test), * p-value < .05, ** p-value < .01.

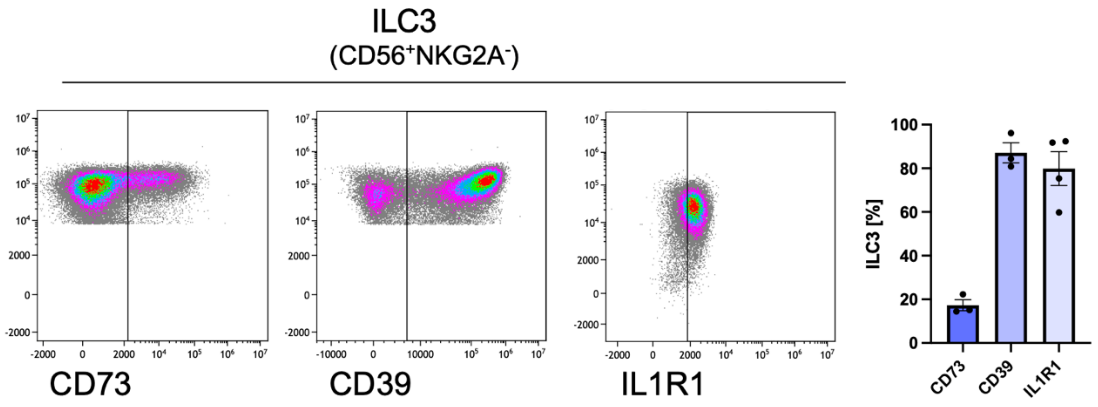

Supplementary Figure 3 | Expression of CD73, CD39, and IL1R1 on *in vitro* ILC3 Cultures were analyzed at day 28 and gated on ILC3 ($CD56^{+}NKG2A^{-}$). Representative dot plots with quantification for cell surface expression of CD73, CD39, and IL1R1 are shown, n=3-4. The heights of the bar graphs represent the mean ± SEM. No levels of significance were calculated as there is no other cell population for comparison.
